# CPNE1 regulates myogenesis through the PERK-eIF2α pathway mediated by endoplasmic reticulum stress

**DOI:** 10.1007/s00441-022-03720-y

**Published:** 2022-12-16

**Authors:** Lin Chen, Ling Pan, Yuexi Zeng, Xiaonan Zhu, Li You

**Affiliations:** Department of Endocrinology and Metabolism, Shanghai General Hospital, Shanghai Jiao Tong University School of Medicine, Shanghai, 200080 China

**Keywords:** Sarcopenia, CPNE1, Endoplasmic reticulum stress, PERK, eIF2α

## Abstract

Sarcopenia is characterized by a progressive reduction in muscle mass or muscle physiological function associated with aging, but the relevant molecular mechanisms are not clear. Here, we identify the role of the myogenesis modifier CPNE1 in sarcopenia. CPNE1 is upregulated in aged skeletal muscles and young skeletal muscle satellite cells with palmitate-induced atrophy. The overexpression of CPNE1 hinders proliferation and differentiation and increases muscle atrophy characteristics in young skeletal muscle-derived satellite cells. In addition, CPNE1 overexpression disrupts the balance of mitochondrial fusion and division and causes endoplasmic reticulum stress. We found that the effects of CPNE1 on mitochondrial function are dependent on the PERK/eIF2α/ATF4 pathway. The overexpression of CPNE1 in young muscles alters membrane lipid composition, reduces skeletal muscle fibrosis regeneration, and exercise capacity in mice. These effects were reversed by PERK inhibitor GSK2606414. Moreover, immunoprecipitation indicates that CPNE1 overexpression greatly increased the acetylation of PERK. Therefore, CPNE1 is an important modifier that drives mitochondrial homeostasis to regulate myogenic cell proliferation and differentiation via the PERK-eIF2α pathway, which could be a valuable target for age-related sarcopenia.

**Supplementary Information:**

The online version contains supplementary material available at 10.1007/s00441-022-03720-y.

## Introduction

Sarcopenia is an accelerated decline in muscle mass that is associated with aging and influenced by physiological factors such as stroke and chronic disease (Cruz-Jentoft and Sayer 2019; Ferri et al. 2020; Su et al. 2020; Wilkinson et al. 2018). In addition to age-related changes in hormone levels, neuromuscular function, and muscle protein turnover, the etiology of sarcopenia is believed to include an elevated pro-inflammatory state and oxidative stress (Can et al. 2016; Liguori et al. 2018). Changes in mitochondrial function, cell proliferation, and endoplasmic reticulum (ER) stress have all been implicated in the progression of sarcopenia with respiratory diseases or age-related sarcopenia (Barreiro et al. 2019; Ferri et al. 2020; Lee et al. 2020; Romanello 2020). For instance, altered membrane lipid composition attributed to differential gene expression was found to promote ER stress and inhibit protein synthesis, which contributes to the loss of muscle mass in sarcopenia (Lee et al. 2020). The epigenetic alteration of gene expression and protein acetylation in skeletal muscle mitochondria have been proposed as underlying mechanisms of ER stress (He et al. 2019; Lee et al. 2020; Tsuda et al. 2018).

Muscle satellite cells are responsible for the regeneration of myoblasts following injury or damage (Bentzinger et al. 2012; Relaix and Zammit 2012). Satellite cells are normally quiescent but on stimulation, they become MyoD1^+^, enter the cell cycle, and generate more satellite cells or MyoD1^+^ myoblasts (Bazgir et al. 2017). In age-related sarcopenia, several genes are known to be differentially expressed in muscle satellite cells such as MuRF1, Glb1, and Atrogin1 (Foletta et al. 2011; Lee et al. 2021). These genes are also used as markers of muscle atrophy and aging (Bodine and Baehr 2015; Lee et al. 2021; Peris-Moreno et al. 2020; Zsofia et al. 2018). In sarcopenia patients with respiratory diseases, expression markers associated with ER stress and the unfolded protein response (UPR), such as protein kinase-like ER kinase (PERK) and activating transcription factors (ATFs), are found to be upregulated in skeletal muscle (Barreiro et al. 2019). Membrane lipid remodeling via the PERK-eukaryotic translation initiation factor 2α (eIF2α) pathway results in ER stress and inhibits the synthesis of proteins (Lee et al. 2020). PERK is believed to be essential for the differentiation of activated satellite cells, whereas eIF2α is associated with the maintenance of the quiescent state (Xiong et al. 2017; Zismanov et al. 2016).

In a recent genome-wide association study, CPNE1 was found to be a modifier of myogenesis associated with appendicular lean mass in adult populations and proposed that CPNE1 could be a suppressor of muscle mass development in humans (Cordero et al. 2019; Tomsig and Creutz 2000). As a soluble membrane-bound protein, CPNE1 may be related to cell signaling and membrane transport pathways. However, the effect and mechanism of CPNE1 on sarcopenia are still unclear. In our study, we investigated the association between CPNE1, ER stress, and mitochondrial function. In particular, we determined whether CPNE1 was involved in the epigenetic manipulation of the PERK/eIF2α/ATF4 pathway through acetylation. Our results demonstrate that CPNE1 is an important modifier that drives mitochondrial homeostasis to regulate myogenic cell proliferation and differentiation via the PERK-eIF2α pathway. CPNE1 could be a valuable target in alleviating the symptoms of sarcopenia.

## Materials and methods

### Animals

Healthy young (3 months old) and old (18 months old) male C57BL/6 mice (Shanghai Laboratory Animal Center Ltd., Shanghai, China) were used in the experiment. The animals were maintained on a 12 h light/dark cycle with ad libitum access to water and food. All animal studies were approved by the appropriate ethics committee and performed in accordance with the ethical standards of Shanghai Jiao Tong University School of Medicine. Fat mass (FM) was determined with an ImpediVET analyzer (ImpediMed, Carlsbad, CA, USA).

### Lipidomic analysis

Lipids were extracted from tibialis anterior (TA) muscles as described previously (He et al. 2019). Briefly, 50 mg of tissue was homogenized with beads in 500 μl 70% methanol. Samples were incubated with 300 μl 70% methanol and 400 μl CHCl_3_ for 10 min at room temperature, centrifuged at 10,000 × g for 10 min, and the lower layer was collected and dried under N_2_. The dried samples were reconstituted in isopropanol: acetonitrile: water (2:1:1) and subjected to triple TOF 5600 mass spectrometry coupled with ultra-performance liquid chromatography (Waters, Milford, MA, USA). Lipids were identified against standards and each lipid species is indicated as a percentage of the total lipids identified. Mass spectrometry (MS) system (SCIEX, Concord, Canada) was used for lipidomic analysis.

### Muscle virus injection

Lentivirus-encoding CPNE1 was injected into the TA muscles of 3-month-old C57BL/6 mice. The TA muscles of mice were injected with 50 µl of lentivirus-encoding CPNE1 (1.3 × 10^7^ unit/µl) or vector (1.3 × 10^7^ unit/µl) once a day for 7 continuous days. TA muscles were harvested 14 days after the last injection.

### Muscle regeneration

Cardiotoxin (CTX) induced muscle injury and regeneration was carried out by injection 50 μl of 10 μM CTX intramuscularly into TA muscle of anesthetized mice using a 1-ml disposable syringe. After injecting 7 and 14 days, muscle tissues were isolated and immediately frozen with liquid nitrogen or fixed in 4% formaldehyde in PBS.

### Grip strength test and hanging grid test

The peak grip force of mice hanging from a grid was measured using a grip strength meter (Muromachi Kikai, Tokyo, Japan). Measurements were performed five times for each mouse, and the maximum values were recorded.

For the hanging grid test, mice were placed in the center of a grid above a pad. The duration of hanging when the grid was inverted upside down was recorded. The data of three independent trials conducted 20 min apart were used for analysis.

### Tetanic force test

The tetanic force test was conducted as described previously (Lee et al. 2020). After mice were euthanized, TA muscles were dissected from the hindlimbs and placed within a force transducer with platinum electrodes and Kreb-Ringer solution. Different force frequencies and increasing stimulation frequencies of 30–200 Hz were made every 500 ms with recovery intervals of 2 min. The level of muscle fatigue was assessed through repeated stimulation for 10 min at a frequency of 1 Hz and 100 V. Data were analyzed using LabChart Pro Software (AD Instruments, Sydney, Australia).

### Hematoxylin–eosin and immunohistochemical staining

TA muscles were collected and fixed in 4% paraformaldehyde and frozen section tissues or paraffin-embedded samples were prepared for subsequently study. Transverse TA muscle tissues were sectioned at 4 μm for hematoxylin and eosin (HE) staining or immunohistochemical (IHC) staining. IHC staining was as described previously (Tang et al. 2018). Sections were incubated in CPNE1 antibody overnight at 4 °C. They were then incubated with biotinylated secondary antibodies for 1 h at room temperature and developed with a DAB Kit (BD Bioscience, San Jose, CA, USA). For slow and fast fibers IHC staining, fast muscle fibers and slow muscle fibers belong to different types of muscle fibers and there are significant differences. First, the diameter of fast muscle fibers is larger than that of slow muscle fibers. Secondly, the vascular network around slow muscle fibers is richer than fast muscle fibers, which also contain more myoglobin and mitochondria than fast muscle fibers. Images were captured using Image-Pro Plus.

### Masson staining

After dewaxing and hydration, slides were sequentially soaked in ponceau magenta for 10 min, 0.2% glacial acetic acid for 1 min, phosphomolybdic acid for 1 min, and 0.2% glacial acetic acid for 1 min to stain the cytoplasm red. Slides were treated with aniline blue for 30 s and soaked with 0.2% glacial acetic acid for 1 min to stain the fibrous tissue blue. The slides were subsequently dehydrated using ethanol, cleared in a dewaxing solution, and mounted. After the acquisition of images, the collagen volume fraction (ratio of blue to red dye area) was calculated using Image-Pro Plus.

### Sirius Red staining

Collagen content was evaluated by Sirius Red staining and colorimetric assay. Slides were dewaxed and hydrated, soaked in Sirius Red dye solution for 1 h, washed with running water for 30 s, dehydrated with ethanol, cleared in a dewaxing solution, and mounted. Images were captured and collagen volume fraction was calculated using Image-Pro Plus.

### Isolation and culture of skeletal muscle-derived satellite cells

TA, gastrocnemius, and extensor digitorum longus muscles of C57BL/6 mice were subjected to 1 mg/ml collagenase for 30 min. Non-muscle tissue was carefully removed and the muscle was minced under a dissection microscope, followed by 30 min incubation. The cell suspension was filtered through a 70-μm nylon filter to obtain a single-cell suspension. Satellite cells were isolated by PE-CD45 − , PE-CD31 − , PE-SCA1 − , PE-CD11b − , Alexa647-α7-Integrin + and PE-CD34 + . Isolated satellite cells were cultured in DMEM containing 10% FBS, 100 IU/mL penicillin, and 100 IU/mL streptomycin. For differentiation, the DMEM contained 2% horse serum, 100 IU/mL penicillin, and 100 IU/mL streptomycin.

### CPNE1 overexpression

The mouse *Cpne1* sequence was amplified by Prime STAR Max DNA polymerase (Takara, Shiga, Japan) using forward CGCAAATGGGCGGTAGGCGTG and reverse TTGGCTGCCCTTTCACTTCC primers. Cells were transfected with *Cpne1* sequence using Lipofectamine 3000 (Invitrogen) according to the manufacturer’s instructions. Lentivirus vector expressing *Cpne1* or negative control were constructed by GenePharma (Shanghai, China). Satellite cells were transduced with 4 µl of lentivirus solution with polybrene (5 µg/ml). 24 h after transduction, the lentivirus containing medium was carefully removed and replaced with fresh medium. Transduced satellite cells were cultured for 3 additional days. Transfection efficiency was confirmed by RT-PCR.

### Wound‐healing assay

The migration ability of the cells was evaluated by a wound-healing assay. Cells were plated in 12‐well plates and grown until 50–60% confluence. A wound was created by scratching the cell monolayer with a sterile 200-μl pipette tip, then rinsed with PBS. The cells were cultured in differentiation medium. The wound area was measured using Image J software at 0 and 24 h.

### Invasion assays

Cell invasion assays were performed using a Transwell chamber (8 μm, Corning, USA). In brief, 100 μl Matrigel was added to a Transwell upper chamber. Then, 200 μl of cells at a density of 4 × 10^5^ cells/mL was added to the upper chamber. After 48 h, the non-adhered cells inside the chamber were rinsed off with PBS and the invaded cells were fixed with ice-cold ethanol for 1 h and then stained with 0.5% crystal violet for 20 min. Three fields were selected randomly and images were captured using an upright microscope.

### Immunofluorescence

Cells were seeded on six-well plates with coverslips and differentiated into myotubes. Myotubes were rinsed in PBS, fixed in 4% paraformaldehyde for 15 min, and then washed three times with PBS and permeabilized in 0.1% Triton X-100 in PBS for 15 min. The myotubes were blocked with 5% BSA for 30 min and then incubated with MyHC, Pax7, MyoD, EdU antibodies overnight at 4 °C, followed by fluorescein isothiocyanate (FITC)‐labeled goat anti‐rabbit IgG secondary antibodies (1:50; Bioss Antibodies, USA) for 1 h at 37 °C. Nuclei were labeled with 4′,6-diamidino-2-phenylindole (DAPI, Beyotime, Shanghai, China) for 5 min. Images were captured under an inverted fluorescence microscope.

### Mitochondrial morphology analysis

Mito-Tracker Green (M7514, Invitrogen, USA) probe was used for mitochondrial morphology observation. Briefly, cells were cultured in confocal dish for 24 h and then incubated with fluorescent mitochondrial probe for 30 min. Olympus FV 1000 laser-scanning confocal microscope was used to obtain mitochondria image.

### Quantitative RT-PCR analysis

Total RNA was isolated from muscle tissues or cells using Trizol reagent (Life Technologies, USA). Quantitative RT-PCR analysis was performed with a 20-μl reaction volume containing cDNA, primers, and SYBR Master Mix (Takara). The primer sequences were listed in Supplementary Table 1. The 2^−△△ct^ method was used to determine the mRNA expression. *Gapdh* as a housekeeping gene*.*

### Western blot analysis

Tissues or cells were lysed with RIPA buffer. Protein content in lysates was measured using bicinchoninic acid and equal amounts of proteins (20 μg) were separated using SDS-PAGE with β-Actin as a loading control. Separated proteins were transferred to PVDF membranes (Millipore, USA) and subsequently blocked with 5% nonfat dry milk. The membranes were incubated overnight at 4 °C with primary antibodies. The following primary antibodies were used: CPNE1 (1:1000, abcam, ab155675), ATROGIN1 (1:1000, Abcam, ab168372), MuRF1 (1:1000, Abcam, ab183094), MyoG (1:1000, Abcam, ab124800), MyoD (1:1000, Abcam, ab133627), GLB1 (1:1000, Abcam, ab203749), p-eIF2α (1:1000, Cell Signaling Technologies, #9721), eIF2α (1:1000, Abcam, ab169528), p-PERK (1:1000, Cell Signaling Technologies, #3179), PERK (1:1000, Abcam, ab229912), ATF4 (1:1000, Abcam, ab31390) and β-Actin (1:1000, Abcam, ab8226). Protein gray values were measured using Image J software.

### Mitochondrial oxygen consumption rate

Oxygen consumption rate (OCR) in 1 mM palmitate treated or CPNE1 transfected satellite cells was measured by using a Seahorse XF Cell Mito Stress Test Kit (Agilent, Santa Clara, USA) and Seahorse XF24 Extracellular Flux Analyzer (Agilent) following the manufacturer’s protocol. The inhibitors (2 μM oligomycin, 1 μM FCCP, and a mixture of 0.5 μM rotenone, and 0.5 μM antimycin) were added at the indicated time points. The experiment was repeated three times and the data showed as the means and SD.

### Detection of reactive oxygen species levels

Reactive oxygen species (ROS) levels were measured by using an ROS Assay Kit (Beyotime). Briefly, satellite cells were incubated with 10 µmol/L DCFH-DA reagent for 20 min, and then cells were washed with PBS for 3 times. The ROS levels were then monitored by Flow cytometry.

### β-galactosidase staining

The β-galactosidase (β-gal) assay was performed with the SA-β-gal staining kit (Sigma). Cells were plated on 12-well plates. After 48 h, cells were incubated with the staining mixture provided by the kit for 16 h at 37 °C. The percentages of β-gal-positive cells were calculated by cell counter in three microscopic fields. Images were captured by an inverted microscope.

### Co-immunoprecipitation assay

To verify the direct binding between CPNE1 and PERK, cells were lysed and incubated with antibodies against CPNE1 (1:1000, abcam, ab155675) at 4 °C overnight. Subsequently, cell lysates were cultivated with protein A/G agarose beads (Santa Cruz, CA, USA) for 2 h. Finally, western blotting with antibodies against PERK (1:1000, Abcam, ab229912) was employed to analyze the respective protein expression in immuno-complexes.

### Statistical analysis

Graphad prism 7.0 and SPSS Statistics 26 were used for statistical analysis. Graphs are presented as mean ± SD, as indicated in the figure legends. Unless otherwise indicated, three independent replicates of each experiment were performed. Significance was calculated by unpaired Student’s *t* tests with two-tailed *p* values and defined as *p* < 0.05.

## Results

### Age-related upregulation of CPNE1 in mice TA muscle

**Fig. 1:**
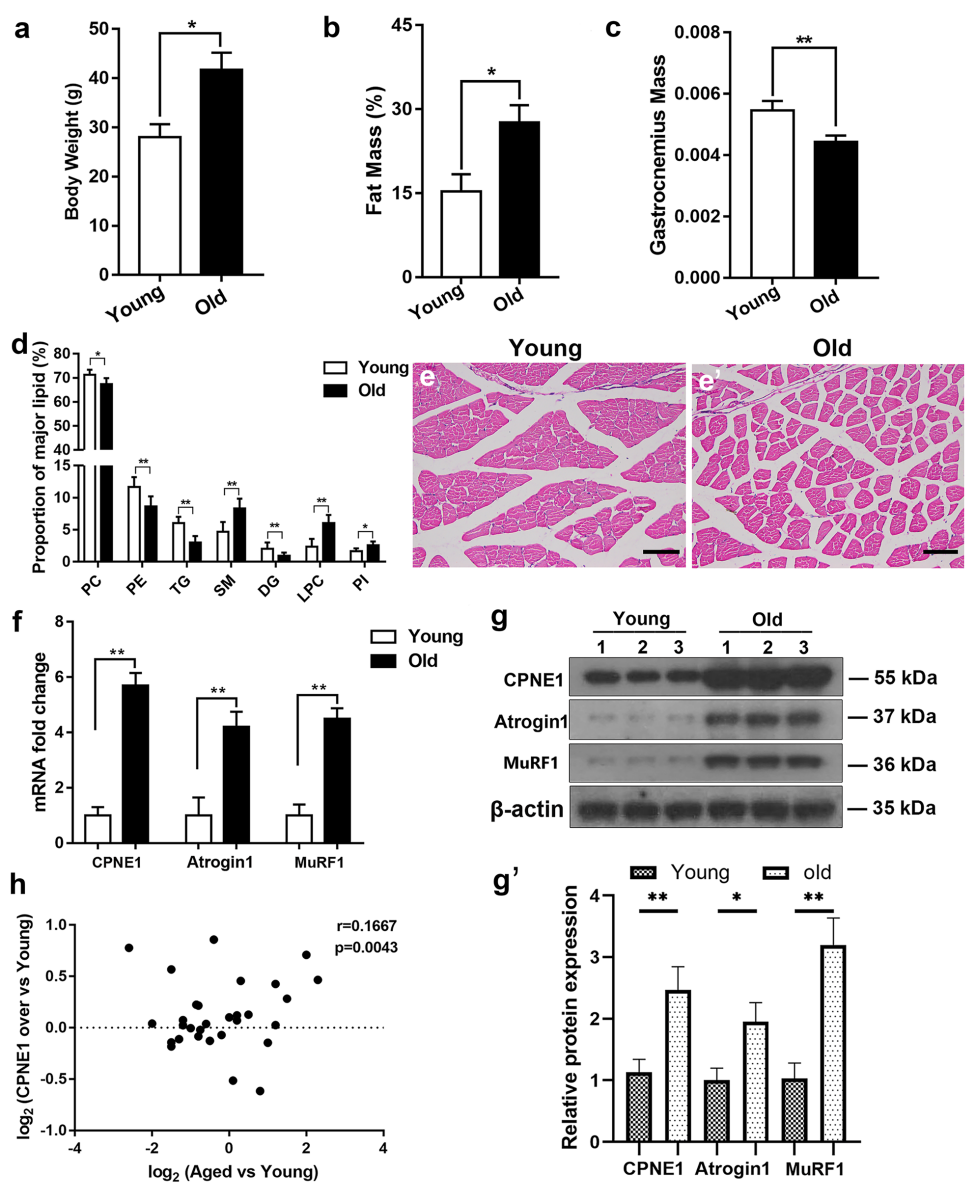
CPNE1 level in young and old mice. **a** Body weight, **b** fat mass, and **c** gastrocnemius mass were examined in young and old muscles. **d** Proportion of major lipid classes in young and old muscles. **e, e**’ HE staining in young and old muscles. Scale bars = 100 μm. CPNE1, Atrogin1, MuRF1 (**f**) mRNA level and (**g, g**’) protein expression in young and old muscles. Protein expression levels were normalized to β-actin. Data in (**h**) were analyzed using Spearman’s correlation between CPNE1 in old and young muscles. ^*^p < 0.05, ^**^p < 0.01. DG, diacylglycerol; LPC, lysophosphatidylcholine; PC, phosphatidylcholine; PE, phosphatidylethanolamine; PI, phosphatidylinositol; SM, sphingomyelin; TG, triglyceride

First, we determined the baseline characteristics of sarcopenia in the tissue of young and old mice. Body weight and FM were significantly higher in older mice than in younger mice, but gastrocnemius mass was lower (Fig. 1a–c). The proportion of major lipid classes was also different between young and old muscles (Fig. 1d). These findings correspond to those found in other studies (Lee et al. 2020). The HE staining of young muscles shows broad and uniformly arranged fibrocollagenous fibers whereas aged muscles have densely packed fibrocollagenous fibers (Fig. 1e). The mRNA expression and protein levels of CPNE1 and the muscle atrophy markers Atrogin1, and MuRF1, were significantly elevated in the tissue of older muscles compared to younger muscles (Fig. 1f–g’). Moreover, the expression of CPNE1 correlated with the age of the muscle tissue (Fig. 1h). Overall, these results confirm that mice with age-related sarcopenia have lower muscle mass with upregulated CPNE1 and atrophy markers, and a variation in lipid composition.

### CPNE1 overexpression increases characteristics of atrophy in young satellite cells

**Fig. 2:**
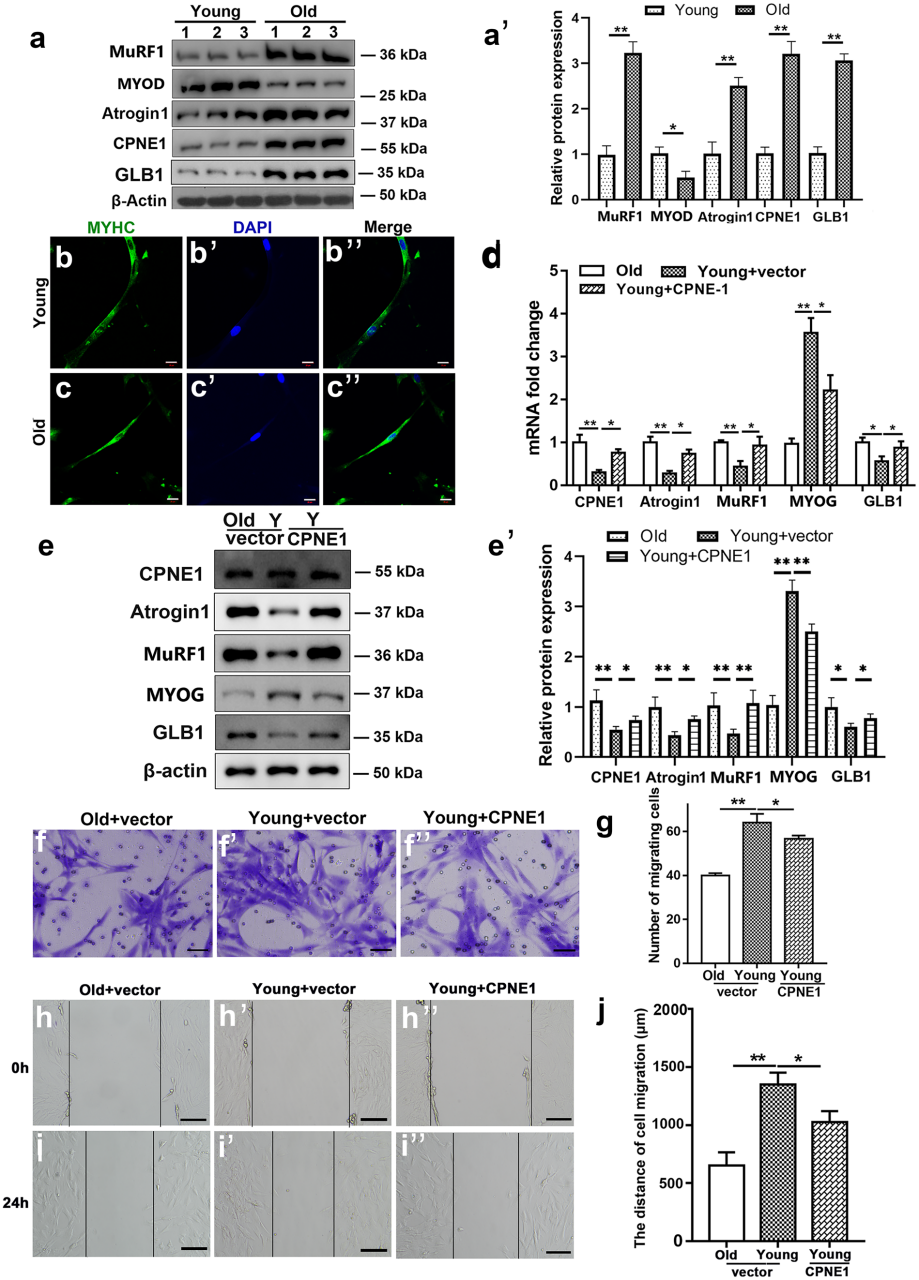
CPNE1 overexpression in young satellite cells Cpne1, Atrogin1, MuRF1 MyoD, and GLB1 (**a, a**’) protein expression in aging and young satellite cells which were transfected with vector or CPNE1 overexpression. Protein expression levels were normalized to β-actin. **b–c**’’ Representative images of myotubes in young and aged satellite cells. Green indicated myosin heavy chain (MyHC) immunofluorescent staining. Blue indicates DAPI stained nuclei. Scale bars = 100 μm. *Cpne1*, *Atrogin1*, *MuRF1*, *MyoG* and *Glb1* (**d**) mRNA level and (**e, e**’) protein expression and quantitative analysis in young and old satellite cells. Protein expression levels were normalized to β-actin. ^*^p < 0.05, ^**^p < 0.01. Representative images (**f–f**’’) and analysis (**g**) of satellite cells wound‐healing with vector or CPNE1 overexpression. Scale bars = 100 μm. Representative images (**h–i**’’) and analysis (**j**) of satellite cells migration with vector or CPNE1 overexpression. Scale bars = 100 μm. ^*^p < 0.05, ^**^p < 0.01

To determine whether CPNE1 influenced other genes differentially expressed during age-related sarcopenia, we measured the protein levels of muscle atrophy markers (Fig. 2a, a’). Satellite cells (CD31-, CD45-, SCA1-, CD11b-, Itga7 + , CD34 +) were isolated by FACS (Fig. S1). *Glb1*, *Cpne1*, *Atrogin1*, and *MuRF1* mRNA expression levels are higher in older satellite cells whereas *MyoG* mRNA levels are lower (Fig. S2b). To verify the difference in young and aged satellite cells differentiation, satellite cells sorted by FACS were induced to differentiate followed by myosin heavy chain (MyHC) staining (Fig. 2b–c’’). CPNE1 overexpression efficiency is tested by RT-PCR (Fig. S2c). β-galactosidase staining demonstrated CRNE1 promoted cellular senescence of young myoblasts (Fig. S2d).

When CPNE1 is overexpressed in young satellite cells, the mRNA levels of *Cpne1*, *Atrogin1*, *MuRF1*, and *Glb1* are significantly increased whereas *MyoG* is significantly lower (Fig. 2d). Similar results were obtained with protein levels (Fig. 2e, e’). The levels of migration were also lower in young satellite cells overexpressing *Cpne1* but still higher than in older satellite cells (Fig. 2f**–**j). Overall, these results indicate that the overexpression of *Cpne1* inhibits the satellite cells migration ability or proliferation of myoblasts.

### CPNE1 inhibits satellite cells differentiation and myotube formation

**Fig. 3:**
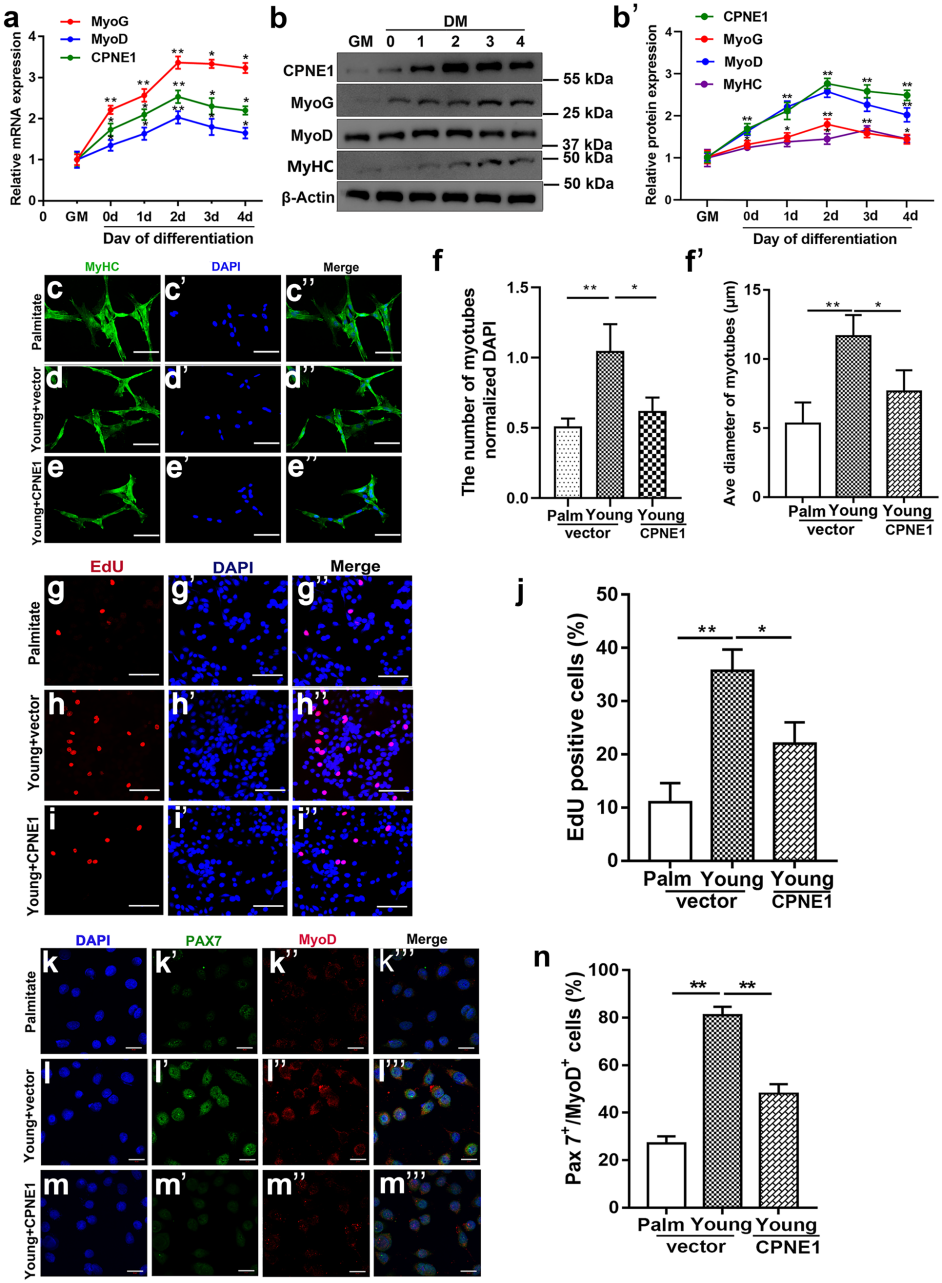
CPNE1 inhibits satellite cells differentiation and myotube formation. **a** qRT-PCR analysis of CPNE1, MyoG, and MyoD expression when satellite cells were cultured in either growth medium for 2 days (GM, proliferating) or differentiation medium for 0, 1, 2, 3, or 4 days (DM 0–4). ^*^p < 0.05, ^**^p < 0.01. **b, b**’ Western blot and quantitative analysis of CPNE1, MyoG, MyoD, and MyHC protein levels when satellite cells were cultured in either GM for 2 days or DM for 0, 1, 2, 3, or 4 days (DM 0–4). Protein expression levels were normalized to β-actin. **c-e**’’ Representative images of myotubes and (**f**) quantitation analysis that the number of myotubes normalized to DAPI stained nuclei from satellite cells with palmitate-induced atrophy or with CPNE1 overexpression. MyHC immunofluorescent staining. Blue indicates DAPI stained nuclei. Scale bars = 100 μm. **f**’’ The average diameter of myotubes. ^*^p < 0.05, ^**^p < 0.01. **g–j** Representative images of EdU staining of satellite cells with palmitate-induced atrophy or CPNE1 overexpression. Scale bar = 100 μm. **F** The number of EdU-positive cells was counted. ^*^p < 0.05, ^**^p < 0.01. **k–m**’’’ Representative immunofluorescence images of Pax7 (green) and MyoD (red) in satellite cells with palmitate-induced atrophy or CPNE1 overexpression. Scale bar = 100 μm. **n** The proportion of proliferating satellite cells (Pax7^+^MyoD^+^).^**^p < 0.01

Palmitate is a free fatty acid that promotes cellular senescence in muscle satellite cells (Chang et al. 2018; Zeng et al. 2010). We compared the effects of *Cpne1* overexpression with palmitate-induced atrophy in satellite cells. In satellite cells transferred from growth to differentiation media, the mRNA expression and protein levels of CPNE1, and MyoD are at the highest level after 2 days and begin to fall thereafter (Fig. 3a**–**b’). MyHC staining indicated that myotube formation was impaired after overexpressing CPNE1 or treated with palmitate, indicating that the overexpression of CPNE1 led to a reduction in myotube differentiation (Fig. 3c–e’’). Moreover, the average myotube diameter was reduced after overexpressing CPNE1 or treated with palmitate (Fig. 3f’). EdU staining assay showed that overexpression of CPNE1 attenuated the proliferation ability in young satellite cells (Fig. 3g–j). The transcription factor PAX7 and the myoblast determination protein MyoD are used to distinguish the proliferative state of satellite cells (Motohashi and Asakura 2014; Von Maltzahn et al. 2013). Pax7 + MyoD − cells are quiescent, Pax7 − MyoD + cells are undergoing myogenic differentiation to generate multinucleated myofibers, and Pax7 + MyoD + cells are proliferating. The proportion of proliferating cells (Pax7 + MyoD +) was lower in satellite cells overexpressing CPNE1 and treated with palmitate (Fig. 3k–n). These results show that overexpressing CPNE1 inhibits satellite cells proliferation and differentiation and induces myotube atrophy.

### CPNE1 overexpression disrupts mitochondrial function and promotes ER stress

**Fig. 4:**
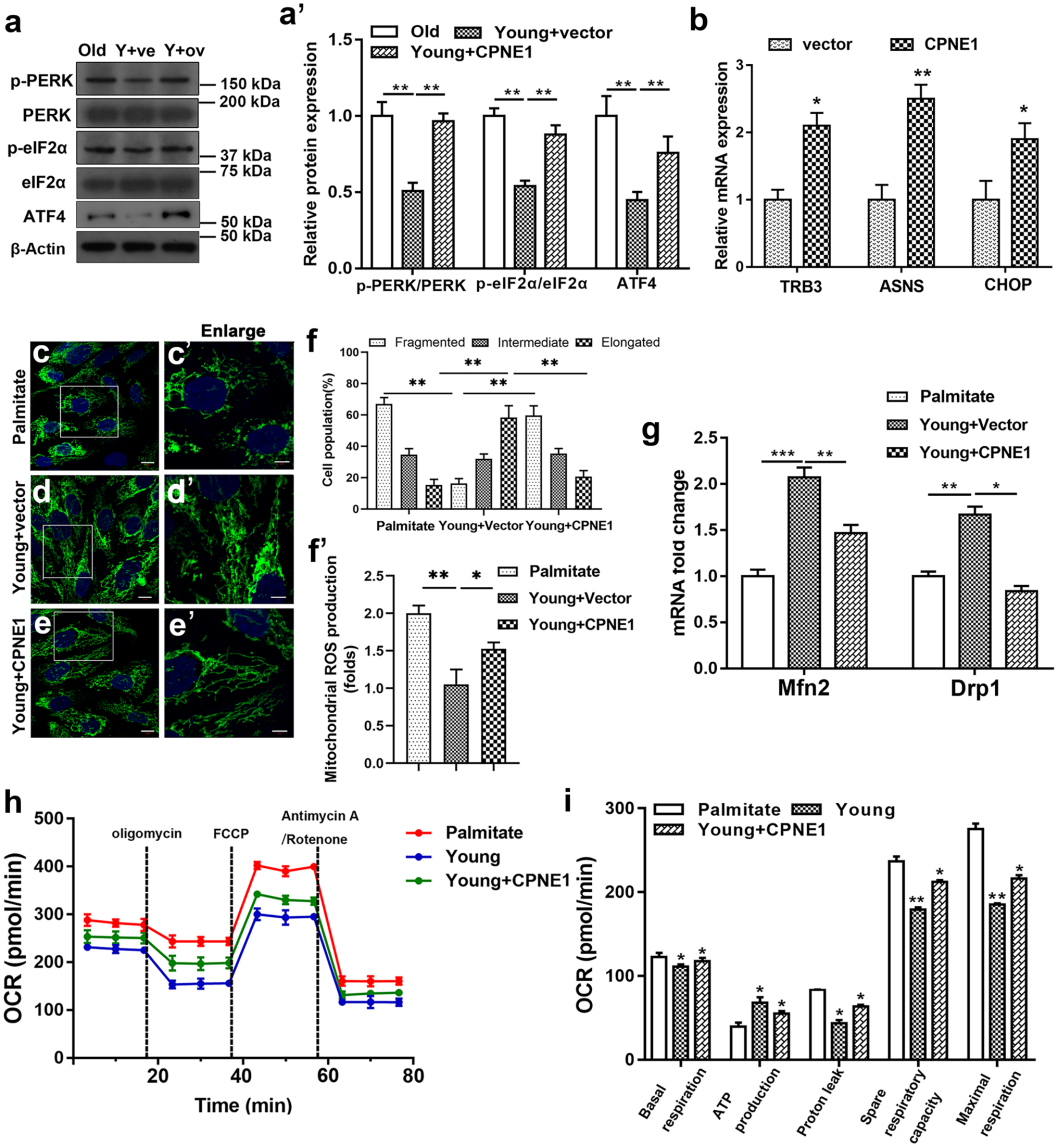
CPNE1 overexpression disrupts mitochondrial function and promotes ER stress. **a** Western blot measurements of p-PERK, PERK, p-eIF2α, eIF2α and ATF4 protein expression and (**a**’) quantitative analysis in satellite cells treated with palmitate or CPNE1 overexpression. Protein expression levels were normalized to β-actin. **b** qRT-PCR results showed that the expression of ATF4 target genes (*Trb3, Asns* and *Chop*) expression after CPNE1 overexpression. **c–e**’ Mitochondrial morphology observation and (**f**) the proportion of cells with elongated, intermediate, and fragmented mitochondria in satellite cells treated with palmitate or CPNE1 overexpression. Scale bar = 100 μm. ^**^p < 0.01. **f**’ ROS levels was determined in satellite cells treated with palmitate or CPNE1 ovexpression. ^*^p < 0.05, ^**^p < 0.01. **g** mRNA level of Mfn2 and Drp1 in satellite cells treated with palmitate or CPNE1 overexpression. ^*^p < 0.05, ^**^p < 0.01. **h** Oxygen consumption rate (OCR) in satellite cells treated with 1 mM palmitate or transfected with CPNE1 overexpression was measured by the XF Cell Mito Stress Test Kit. The data was showed as the mean ± SD (*n* = 3). **i** Basal respiration, ATP production, proton leak, spare respiratory capacity, and maximal respiration in satellite cells treated with palmitate or transfected with CPNE1 overexpression. The data was showed as the mean ± SD (*n* = 3). ^*^p < 0.05, ^**^p < 0.01

Phosphorylation of PERK and eIF2α participate in the differentiation of satellite cells and are markers used to evaluate ER stress (Guangyan et al. 2017; Oslowski and Urano 2011; Zismanov et al. 2016). We observed the levels of PERK and eIF2α phosphorylation in satellite cells with CPNE1 overexpressed. The level of p-PERK, p-eIF2α and ATF4 was higher in older satellite cells and young satellite cells overexpressing CPNE1 than in young satellite cells (Fig. 4a, a’). qRT-PCR results showed that the expression of ATF4 target genes (*Trb3, Asns* and *Chop*) increased after CPNE1 overexpression (Fig. 4b). These results suggest that the PERK/eIF2α/ATF4 axis was indeed activated upon CPNE1 overexpression. As shown in Fig. 4c–f, extensive mitochondrial elongation was observed in the young satellite cells, whereas significant fragmentation of mitochondria was observed in palmitate-induced satellite cells and overexpressing CPNE1 satellite cells, suggesting a disruption of the balance between mitochondrial fusion and fission. The production of mitochondrial ROS was significantly increased in palmitate-induced satellite cells and overexpressing CPNE1 satellite cells, significantly inhibited in young satellite cells (Fig. 4f’). MFN2 and DRP1 are proteins involved in mitochondrial fusion and fission, respectively (Rodrigues and Ferraz 2020). We found that mitochondrial fusion and fission (*Mfn2* and *Drp1)* mRNA expression were reduced in satellite cells treated with palmitate or overexpressing CPNE1 (Fig. 4g), which suggests that overexpression of CPNE1 attenuates mitochondrial fission and fusion. We then measured the difference in OCR in young satellite cells compared with those overexpressing CPNE1 or treated with palmitate. Basal respiration, proton leaks, spare respiratory capacity, and maximal respiration were higher in palmitate treatment or overexpressing CPNE1 (Fig. 4h–i). However, ATP production was higher in young satellite cells than in satellite cells overexpressin*g* CPNE1 or treated with palmitate (Fig. 4h–i). Overall, our results indicate that CPNE1 upregulates the PERK/eIF2α/ATF4 pathway and disrupts the balance of mitochondrial fusion and division.

### Acetylation of PERK is increased by the overexpression of CPNE1

**Fig. 5:**
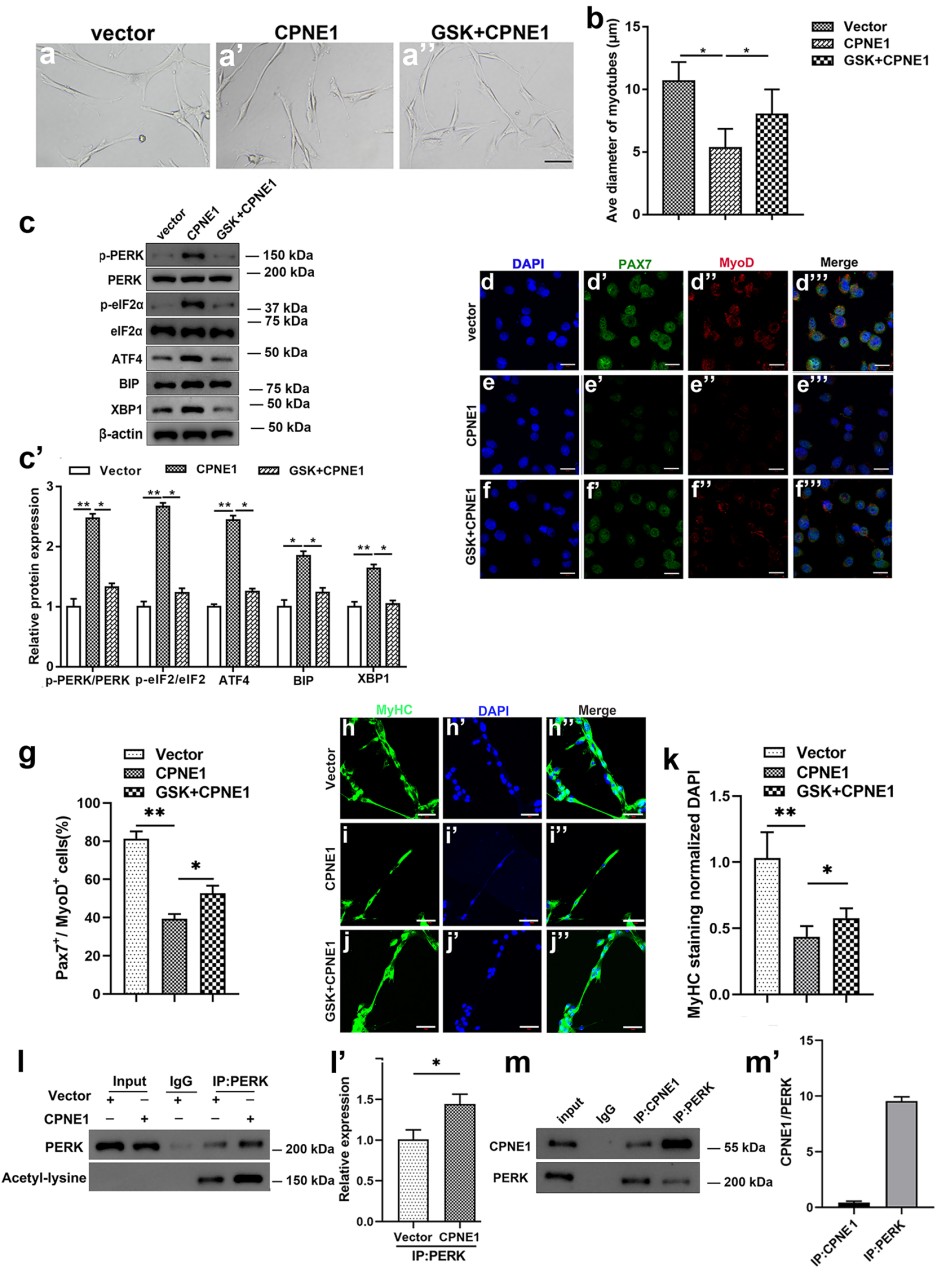
Acetylation of PERK is increased by the overexpression of CPNE1. **a, a**’’ Representative images of myotubes in satellite cells with CPNE1 overexpression or PERK inhibitor GSK2606414 (5 μmol/L). **b** The average diameter of myotubes. ^*^p < 0.05, ^**^p < 0.01. **c** Western blot and (**c**’) quantitative analysis for p-PERK/ERK, p-eIF2α/eIF2α, ATF4, BIP, and XBP1 when satellite cells were transfected with CPNE1 or treated with GSK2606414. Protein expression levels were normalized to β-actin. ^*^p < 0.05, ^**^p < 0.01. **d–f**’’ Representative immunofluorescence images of Pax7 (green) and MyoD (red) and (**g**) the proportion of proliferating satellite cells (Pax7^+^MyoD^+^) in satellite cells transfected with CPNE1 expression vector or GSK2606414. Scale bar = 100 μm. ^*^p < 0.05, ^**^p < 0.01. **h–j**’’ Representative immunofluorescence images of MyHC (green) and DAPI (blue) and (**k**) quantitative analysis in satellite cells transfected with CPNE1 expression vector or GSK2606414. Scale bar = 100 μm. ^*^p < 0.05, ^**^p < 0.01. **i, i**’ Satellite cells were transfected with empty vector or CPNE1 vector. PERK was immunoprecipitated, and the level of acetylation was determined by immunoblotting with an anti-acetyl-lysine antibody. **m, m**’ Physical interaction between endogenous CPNE1 and PERK measured by co-immunoprecipitation

To further investigate the consequence of the PERK/eIF2α/ATF4 pathway upregulation and the effects this has on myotube formation, we assessed the differential expression when CPNE1 is overexpressed or/and the inhibition of PERK on myotube formation. Figure 5a, a’’ shows representative images of myotubes from satellite cells, in which myotubes were inhibited after CPNE1 overexpression and reversed by the PERK inhibitor GSK2606414. The average diameter of the myotubes was significantly lower in satellite cells with CPNE1 overexpressed and was increased when combined with GSK2606414 treatment (Fig. 5b). Interestingly, results showed that p-PERK, p-eIF2α, ATF4, BIP, and XBP1 protein levels were increased when CPNE1 was overexpressed but decreased by together with GSK2606414 treatment (Fig. 5c, c’). Representative immunofluorescence images of Pax7, MyoD (Fig. 5d–g) and MyHC (Fig. 5h–k) indicated that the levers of Pax7, MyoD, and MyHC of satellite cells were impaired when CPNE1 transfection alone or combined with GSK2606414 treatment. Satellite cells transfected with vector or CPNE1 were immunoprecipitated with PERK after treating satellite cells with acetyl-lysine (Fig. 5i, i’). A physical interaction was confirmed between endogenous CPNE1 and PERK by co-immunoprecipitation (Fig. 5m, m’). These results indicate that CPNE1 overexpression greatly increases the acetylation of the ER stress-related protein PERK, which inhibits the proliferation and differentiation of satellite cells.

### Increased levels of ER stress in TA muscles overexpressing CPNE1

**Fig. 6:**
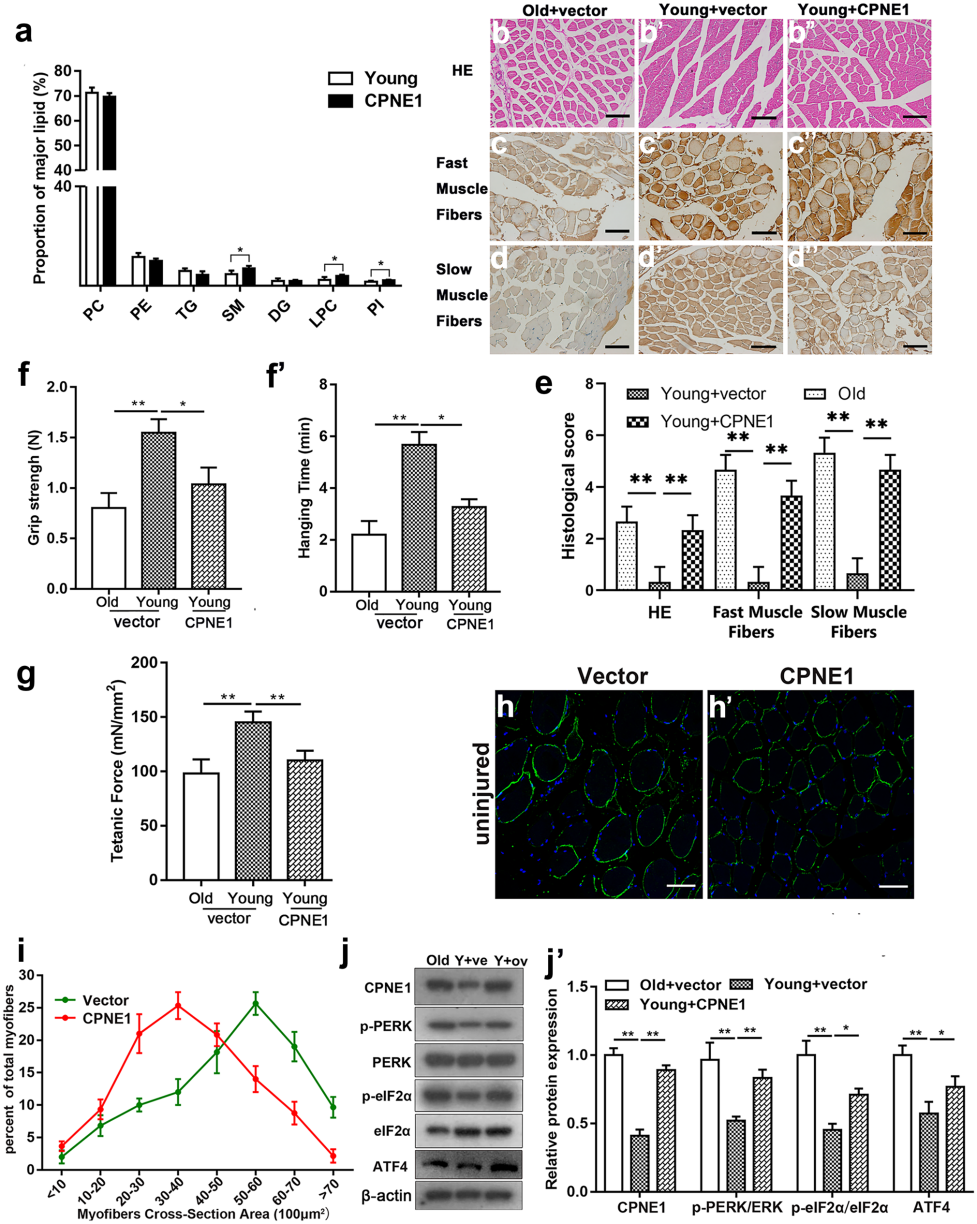
Increased levels of ER stress in TA muscles overexpressing CPNE1. **a** Proportion of major lipid classes in muscles injected with control or CPNE1 vector. **b**–**e** Hematoxylin–eosin (HE) and IHC staining and histological score of fast and slow myosin heavy chain. The staining area was graded as 0 (< 5%), 1 (5–25%), 2 (25–50%), 3 (50–75%), and 4 for (> 75%). The staining intensity was scored as 0 (negative), 1 (weak), 2 (medium), and 3 (strong). The staining area and intensity percentage were added together to make a total histological score. Scale bar = 100 μm. ^**^p < 0.01. (**f**) Grip strength, (**f**’) hanging time, (**g)** tetanic force in muscles injected with control vector or CPNE1 expression vector. **h, h**’ Immunofluorescence assay for laminin (green) and nuclei (blue) on regenerating TA muscle cross-sections when CPNE1 was overexpressed. Scale bar = 100 μm. **i** Distribution of myofiber diameters of TA muscles after mice were transfected control vector or CPNE1 overexpression (*n* = 3). Western blot measured p-PERK, PERK, p-eIF2α, eIF2α and ATF4 (**j**) protein expression and (**j**’) quantitative analysis in control vector or CPNE1 overexpression. Protein expression levels were normalized to β-actin. ^*^p < 0.05, ^**^p < 0.01

To determine the influence of CPNE1 on muscle atrophy in vivo, lentivirus-encoding CPNE1 or a vector control was injected into the TA muscles of the young mice for 7 days. The proportion of major lipids was different between young and old tissue with SM, LPC, and PI showing a significant increase (Fig. 6a). HE staining showed that the overexpression of CPNE1 in young TA muscles promoted the degeneration that was observed in older tissues (Fig. 6b–d’’). This was confirmed by the staining of the fast and slow MyHC with IHC. The overexpression of CPNE1 diminished the intense staining of younger tissues (Fig. 6e). Moreover, grip strength, hanging time, and tetanic force in the young mice were all weakened by the overexpression of CPNE1 in TA muscles (Fig. 6f–g). Laminin is distributed in the basal lamina structure of muscle fibers and is an important and biologically active part of the basal lamina, affecting cell differentiation, migration, and adhesion. Laminin staining judged the size of muscle fibers by the cross-section of muscle fibers, and our results showed that muscle fiber size was reduced after overexpression of CPNE1, suggesting that high levels of CPNE1 induce muscle atrophy (Fig. 6h, h’). The distribution of myofiber diameters in TA muscles also differed between the young mice transfected with CPNE1 and those transfected with vector. The size of the muscle fibers with CPNE1 overexpressed was significantly smaller than vector (Fig. 6i). ER stress, which was indicated by the upregulation of the p-PERK, p-eIF2α, and ATF4 protein expression, was also more severe in the TA tissues of old mice and young mice overexpressing CPNE1 than in the young control mice (Fig. 6j, j’). Overall, these results indicate that the overexpression of CPNE1 results in the in vivo weakening of muscle strength and increased ER stress in young mice.

### Tissue regeneration is impaired by the overexpression of CPNE1 in mice TA muscles

**Fig. 7:**
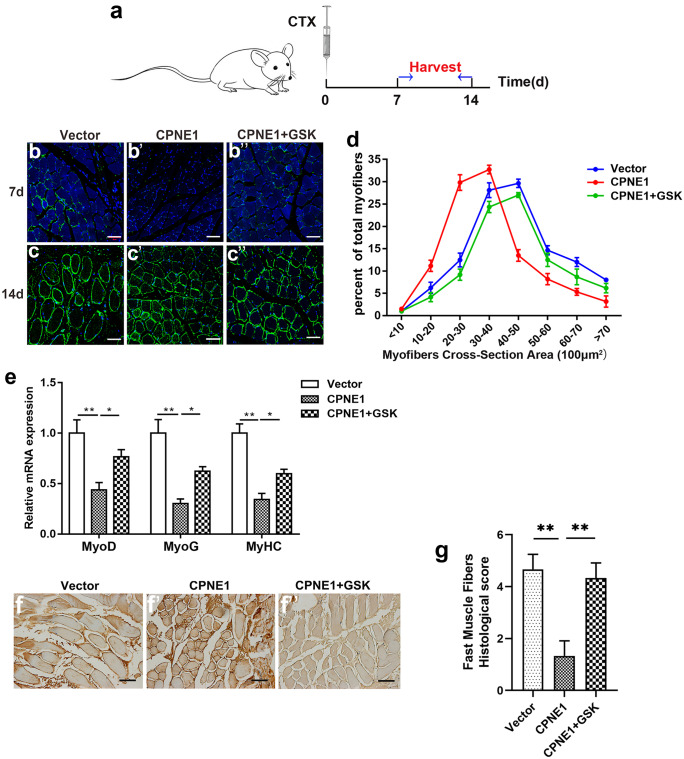
Tissue regeneration in mice TA muscles overexpressing CPNE1. **a** Schematic of cardiotoxin (CTX) injected into the TA muscles, harvested, and analyzed at 7 days and 14 days post-injury. **b–c**’’ Immunofluorescence assay for laminin (green) and nuclei (blue) in regenerating TA muscle cross-sections at 7 days and 14 days. Scale bar = 200 μm. **d** Distribution of myofiber diameters of CTX-injured TA muscles after mice were transfected with CPNE1 or CPNE1 together with GSK2606414 at 14 days after injury (*n* = 3). **e** Relative mRNA expression of MyoD, MyoG and MyHC after mice transfect CPNE1 overexpression or together with GSK2606414 at 7 days and 14 days. **f, g** IHC staining and histological score of TA muscle tissue after overexpression of CPNE1 or together with GSK2606414 treatment. Scale bar = 100 μm. ^**^p < 0.01

TA muscles of mice were injected with the cardiotoxin (CTX) to induce an acute muscle injury (Garry et al. 2016). Cross-sections were assessed using immunofluorescence at 7 and 14 days post-injury (Fig. 7a–c’’). Laminin was used to identify satellite cells in the muscle tissues. Satellite cells are activated to regenerate and repair injured muscle. After overexpression of CPNE1, the size of muscle fibers was smaller than vector but recovered after PERK inhibitor GSK2606414 treatment (Fig. 7b–c’’), the effects of CPNE1 overexpression were intensified. The distribution of myofiber diameters of CTX-injured TA muscles after transfection with CPNE1 or CPNE1 together with GSK2606414 at 14 days after injury was shown in Fig. 7d. CPNE1-overexpressing myofiber size was significantly smaller than vector but increased after PERK inhibitor treatment (Fig. 7d). The relative mRNA expression of *MyoD*, *MyoG* and *MyHC* decreased after overexpression of CPNE1, but increased after combined with GSK2606414 treatment (Fig. 7e). IHC staining confirmed that the overexpression of CPNE1 lowered levels of fast myosin heavy chain in TA muscle tissue but reversed by GSK2606414 (Fig. 7f, g). Our results indicate that the overexpression of CPNE1 hinders TA muscle proliferation and differentiation in vivo and increases muscle atrophy, however, this phenotype is reversed by PERK inhibitor.

## Discussion

At present, sarcopenia has few effective pharmacological interventions but various molecular pathways associated with the condition are beginning to emerge (Feike et al. 2021; Waltz et al. 2017). The UPR and ER stress have been implicated in the development of age-related sarcopenia, with the PERK/eIF2α pathway playing a predominant role (Caterina et al. 2017; Hart et al. 2019; Romanello and Sandri 2021). In this study, we have examined interactions between the PERK/eIF2α pathway and CPNE1. Overall, our findings indicate that ER stress activation of the PERK-eIF2α pathway leads to the regulation of age-related skeletal satellite cells migration and differentiation through CPNE1. To arrive at this conclusion, we have assessed proliferation and differentiation in satellite cells, muscle atrophy mitochondrial fusion and division, endoplasmic reticulum stress, and acetylation.

The involvement of CPNE1 in sarcopenia is supported by the findings of a genome-wide association study of appendicular lean mass in mice and humans (Cordero et al. 2019). The differential expression of CPNE1 is more often associated with poor prognosis in diseases such as cancer and diabetes (Azarova et al. 2021; Liang et al. 2017; Shunlin et al. 2018). However, sarcopenia is also associated with these diseases and there may be a correlation between CPNE1 expression, disease progression, and muscle atrophy. Atrogin1 and MuRF1 are well-known markers of sarcopenia and their upregulation is also observed in other diseases (Liu et al. 2017; Rom and Reznick 2016). Their suppression has been found to alleviate muscle loss in diabetes and dexamethasone-induced myotube atrophy (Castillero et al. 2013; Liu et al. 2017).

To determine whether CPNE1 is a key regulator in muscle atrophy, we assessed the characteristics of sarcopenia in young skeletal muscle-derived satellite cells when CPNE1 is overexpressed. The overexpression of CPNE1 in satellite cells inhibited proliferation and differentiation. Further investigations established that CPNE1 disrupted the balance of mitochondrial fusion and division and causes ER stress. An association between mitochondrial dysfunction and ER stress in the development of muscle atrophy has been proposed in several studies (Leduc-Gaudet et al. 2021). We found less mitochondrial fusion and fission in satellite cells overexpressing CPNE1 than in young control satellite cells. OCR was also higher in satellite cells overexpressing CPNE1 than in young satellite cells. This reiterates results obtained from elderly patients and old and young mice models, where levels of mitochondrial biogenesis, fission/fusion, autophagy, and the respiratory capacity of mitochondria isolated from TA muscles decline through aging (Annunziata et al. 2018; Son et al. 2017).

After establishing a connection between ER stress and characteristics of sarcopenia, we determined whether the impact of CPNE1 overexpression on mitochondrial function in young satellite cells was influenced by the PERK/eIF2α/ATF4 pathway, which is associated with ER stress and the UPR (Zito 2019). We found that CPNE1 overexpression impeded myotube formation and was reversed by PERK inhibitor. Moreover, we found an interaction between CPNE1 and PERK involving acetylation. Overexpression of CPNE1 increases the acetylation of PERK, which in turn inhibits the proliferation and differentiation of satellite cells. In addition to CPNE1, other proteins are believed to regulate the acetylation of PERK (Zhang et al. 2021). For instance, in a mouse model of chronic kidney disease, ER stress-induced vascular calcification was found to depend on PERK acetylation (Zhang et al. 2021). Terpinen-4-ol could alleviate vascular calcification in these mice by inhibiting the PERK/eIF2α/ATF4 pathway through the SIRT1 deacetylation of PERK.

We also investigated the overexpression of CPNE1 in the TA muscles of young mice. The major lipid species found in the skeletal muscle of young mice overexpressing CPNE1 were comparable to that found in old mice. In our study, CPNE1 overexpression in young muscles reduced muscle regeneration and the exercise capacity of the mice. Muscles in the mice overexpressing CPNE1 were weakened and were reversed by the PERK inhibitor, signifying that CPNE1 is an important modifier that drives mitochondrial homeostasis to regulate myogenic cell proliferation and differentiation via the PERK-eIF2α pathway.

To conclude, we have identified CPNE1 as a myogenesis modifier in sarcopenia. CPNE1 is upregulated in old skeletal muscles and young skeletal muscle satellite cells with palmitate-induced atrophy. The overexpression of CPNE1 hinders proliferation and differentiation and increases muscle atrophy in young satellite cells and disrupts the balance of mitochondrial fusion and division to cause ER stress. The effects of CPNE1 on mitochondrial function are dependent on the PERK/eIF2α/ATF4 pathway. The overexpression of CPNE1 in young muscles alters membrane lipid composition, reduces skeletal muscle fibrosis regeneration, and exercise capacity in mice. Therefore, CPNE1 could be a valuable target for age-related sarcopenia.

## Supplementary Information

Below is the link to the electronic supplementary material.

Supplementary file1 (TIF 38372 KB)

Supplementary file2 (DOCX 13 KB)

Supplementary file3 (TIF 145 KB)

Supplementary file4 (TIF 3146 KB)

Supplementary file5 (DOCX 14 KB)

Supplementary file6 (TIF 33202 KB)

Supplementary file7 (TIF 42077 KB)

Supplementary file8 (TIF 26519 KB)

Supplementary file9 (TIF 31266 KB)

Supplementary file10 (TIF 44847 KB)

## Data Availability

The data of this study are available from the corresponding author upon reasonable request.
