# Supplementary figures and images for: CPNE1 regulates myogenesis through the PERK-eIF2α pathway mediated by endoplasmic reticulum stress
Source: Cell Tissue Res. 2022 Dec 16;391(3):545–60. doi: 10.1007/s00441-022-03720-y (PMC9974702; doi:10.1007/s00441-022-03720-y)

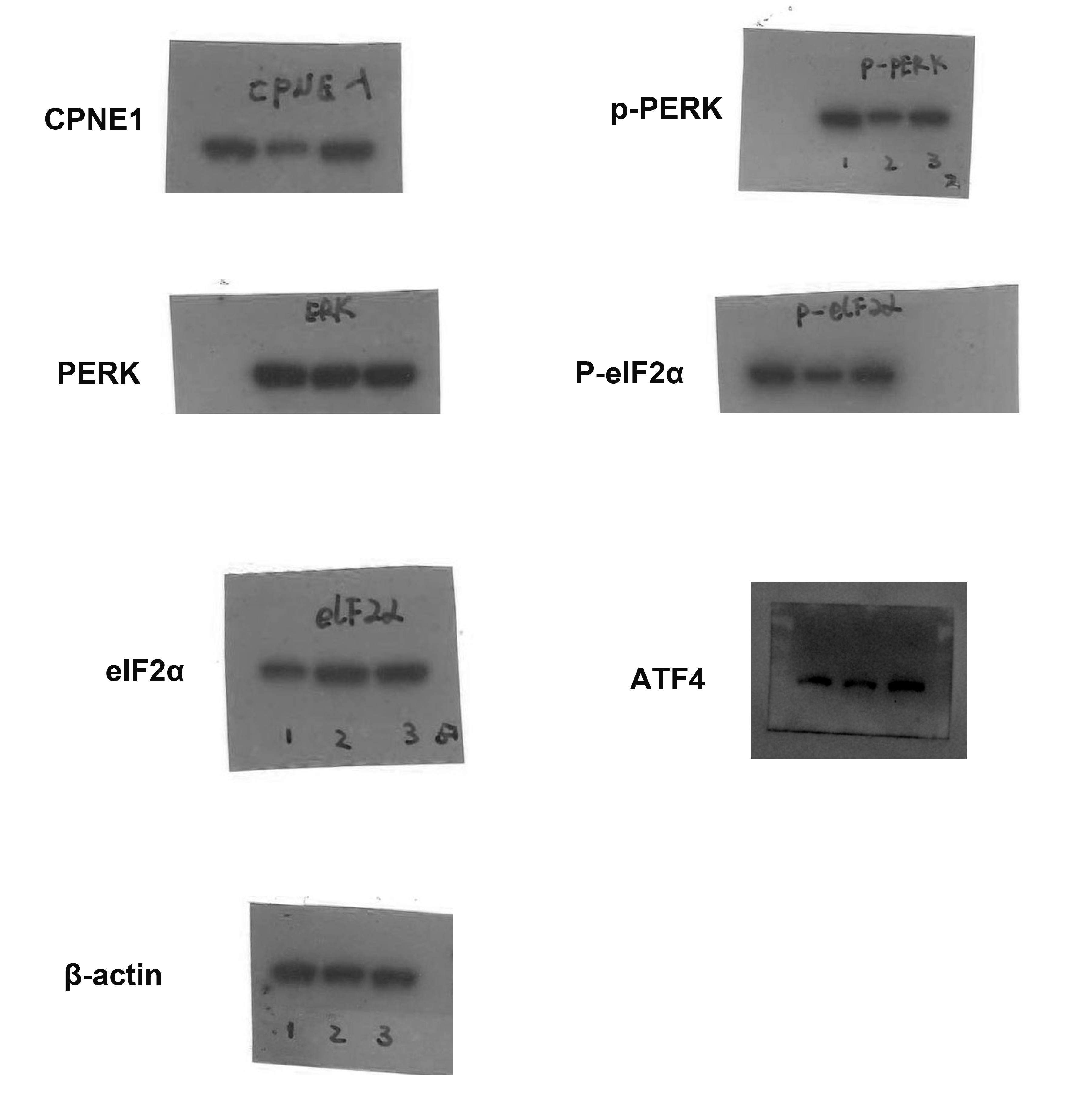

Supplement: Supplementary file 1 — Supplementary file1 (TIF 38372 KB) [file 441_2022_3720_MOESM1_ESM.tif]

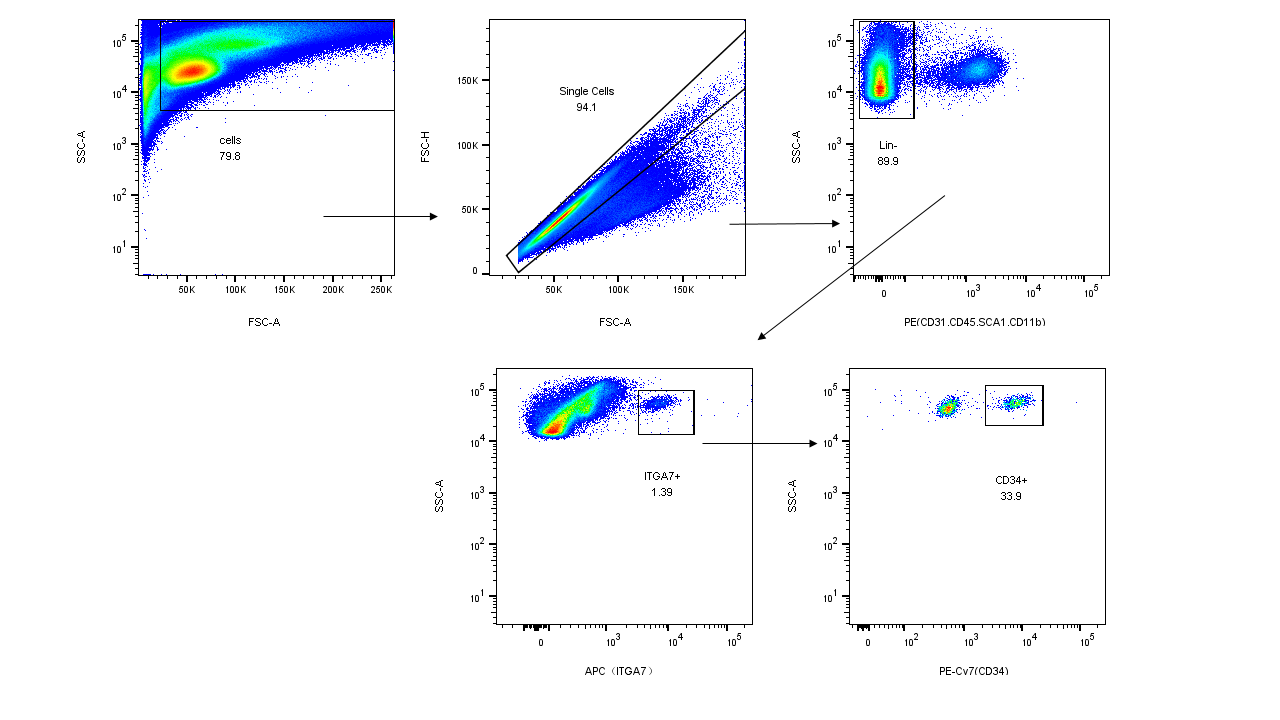

Supplement: Supplementary file 3 — Supplementary file3 (TIF 145 KB) [file 441_2022_3720_MOESM3_ESM.tif]

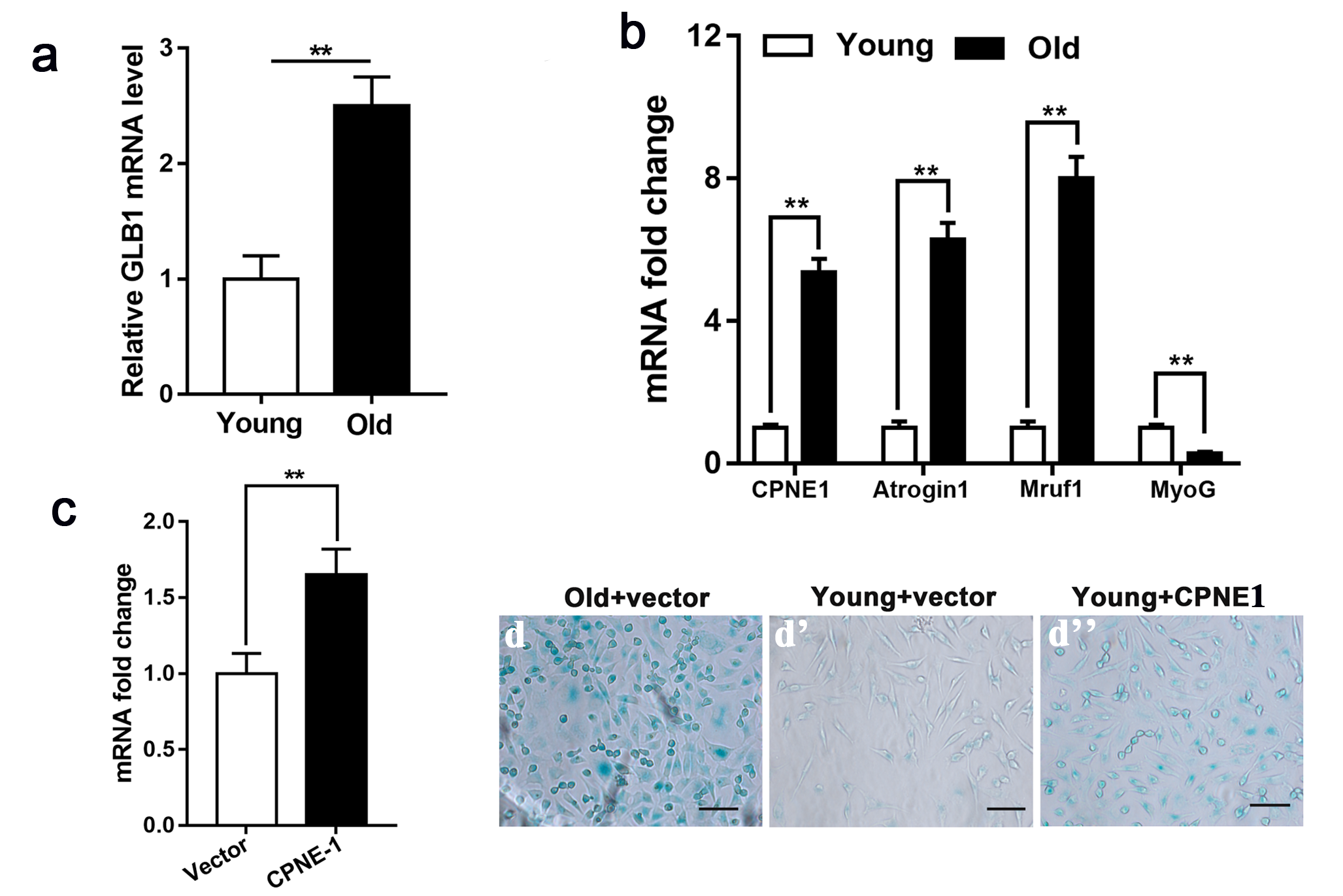

Supplement: Supplementary file 4 — Supplementary file4 (TIF 3146 KB) [file 441_2022_3720_MOESM4_ESM.tif]

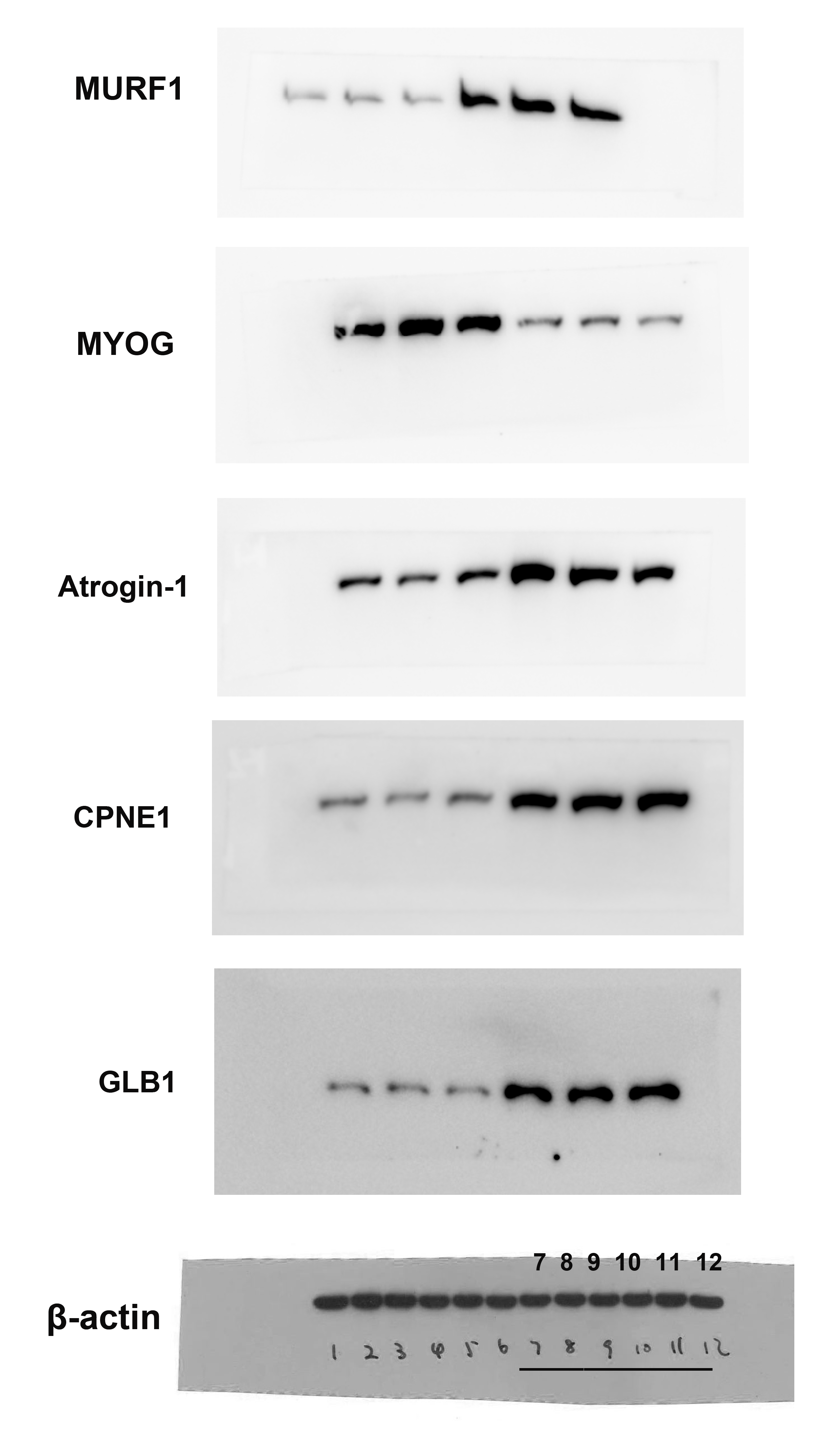

Supplement: Supplementary file 6 — Supplementary file6 (TIF 33202 KB) [file 441_2022_3720_MOESM6_ESM.tif]

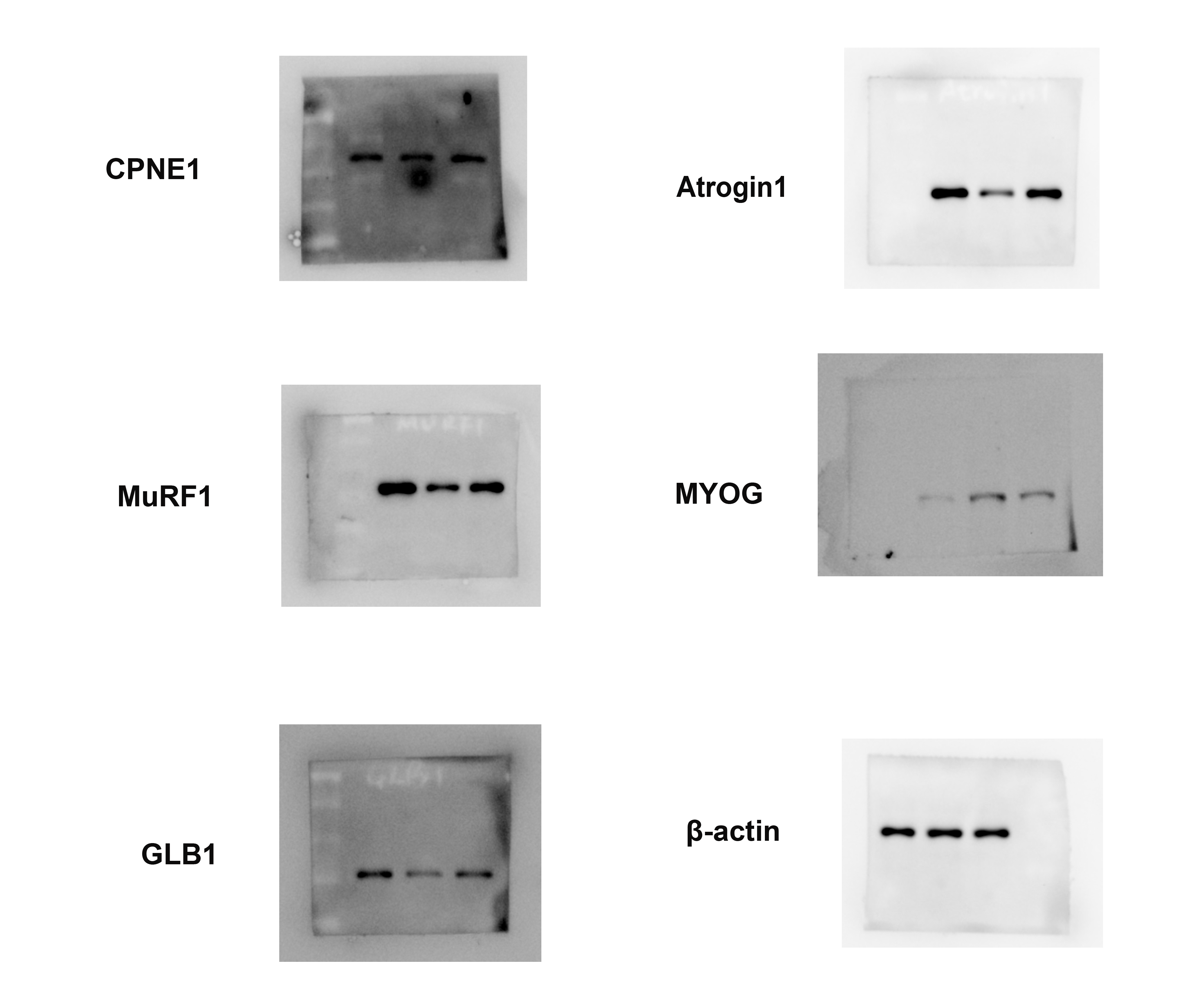

Supplement: Supplementary file 7 — Supplementary file7 (TIF 42077 KB) [file 441_2022_3720_MOESM7_ESM.tif]

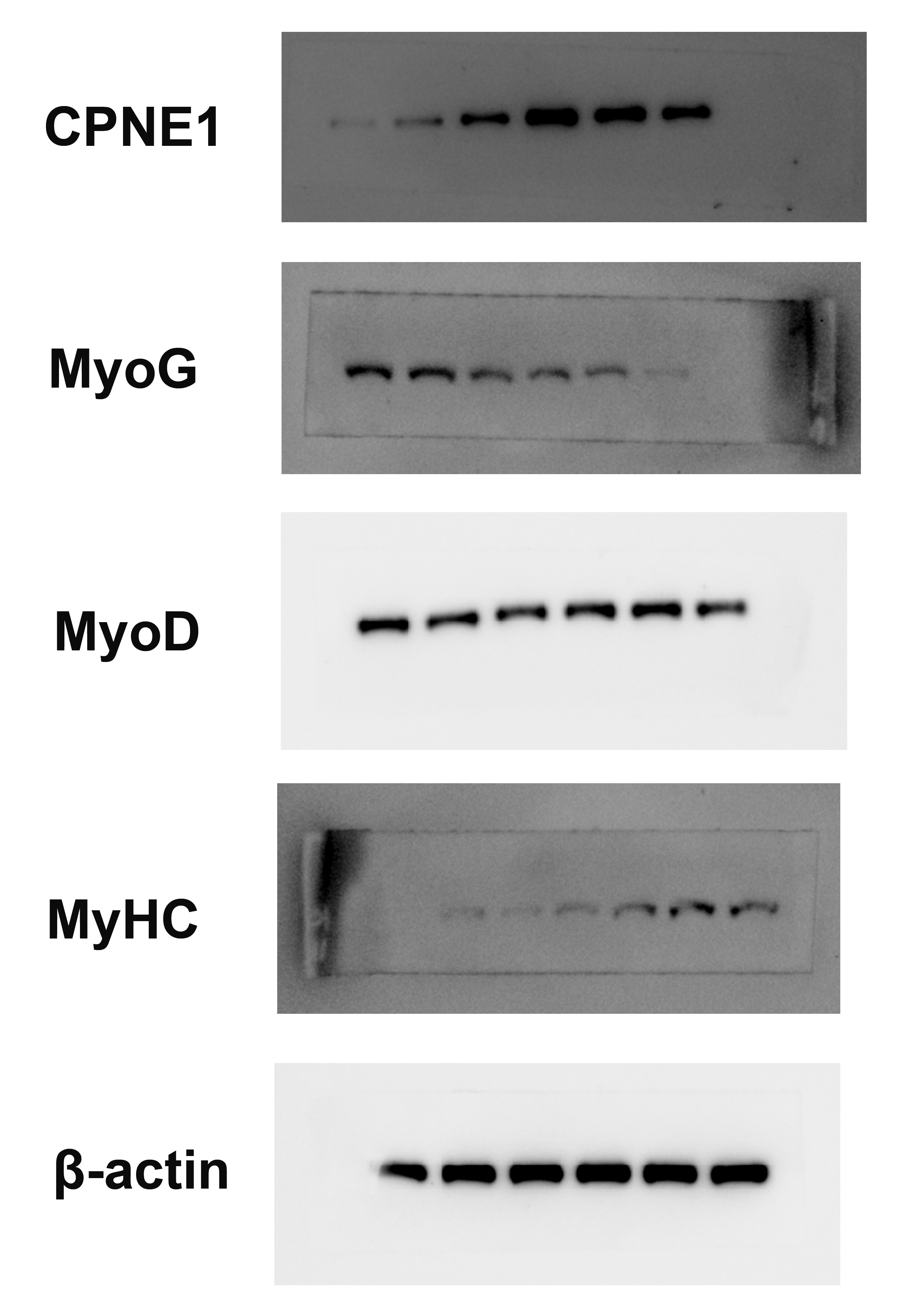

Supplement: Supplementary file 8 — Supplementary file8 (TIF 26519 KB) [file 441_2022_3720_MOESM8_ESM.tif]

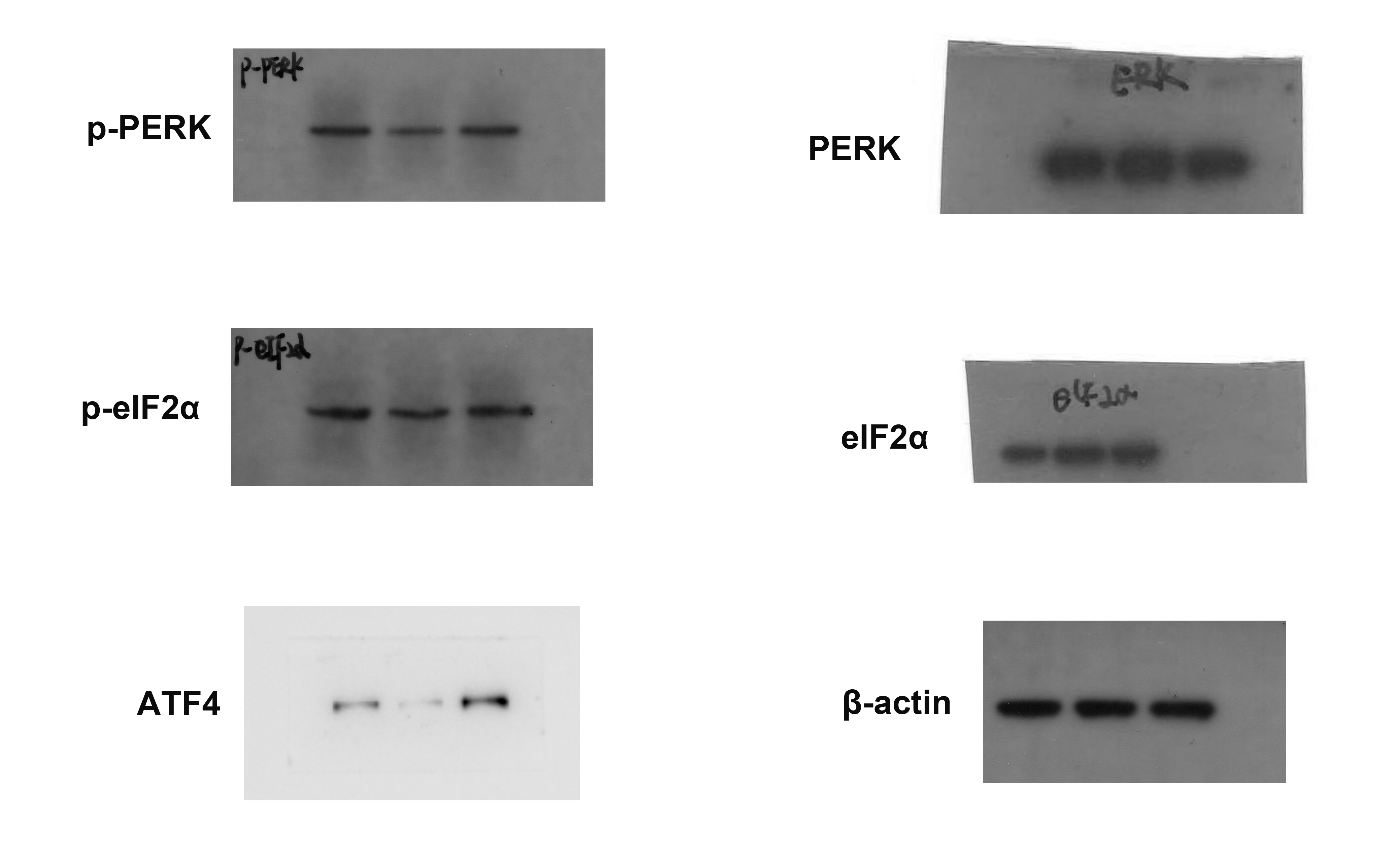

Supplement: Supplementary file 9 — Supplementary file9 (TIF 31266 KB) [file 441_2022_3720_MOESM9_ESM.tif]

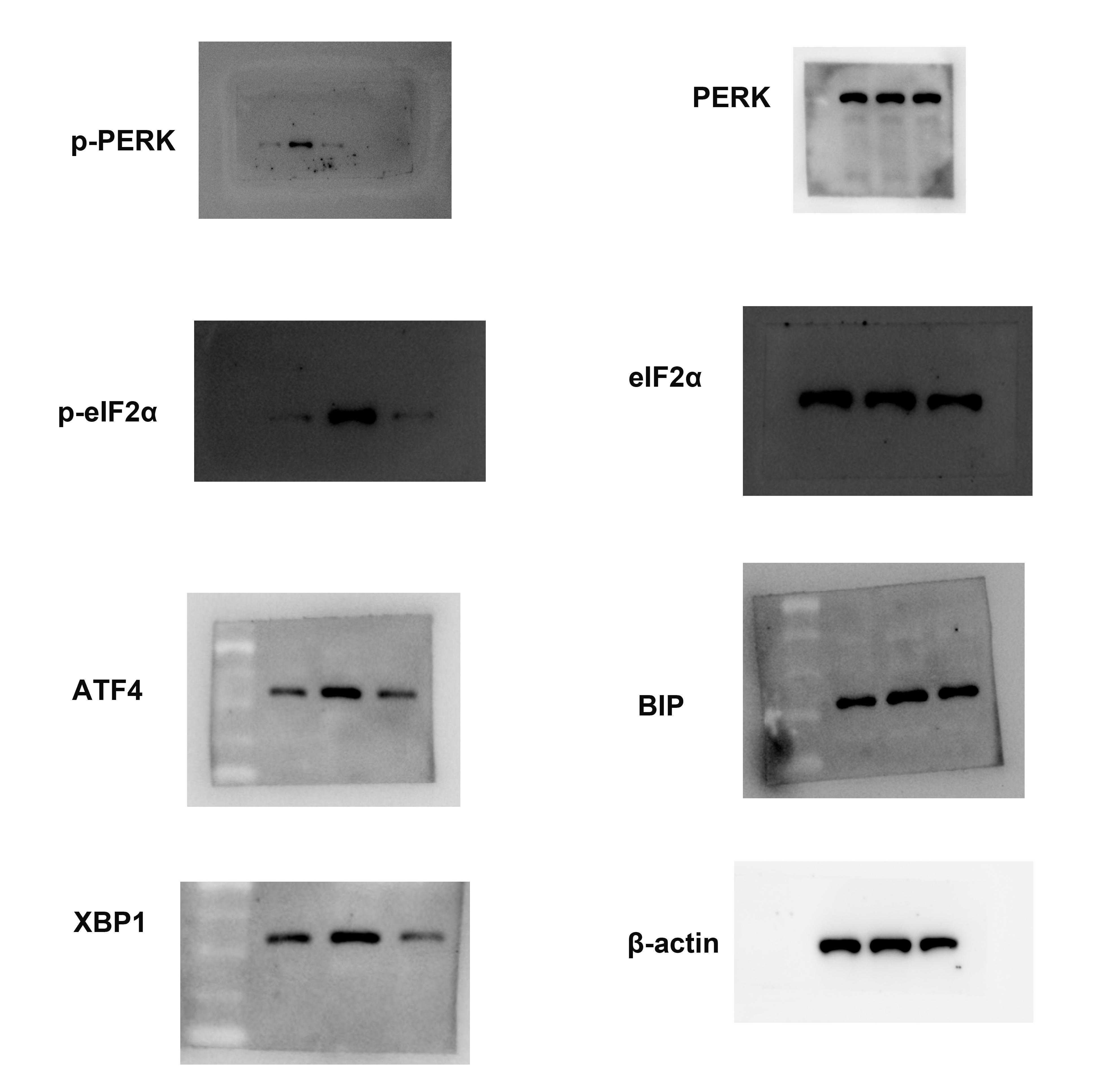

Supplement: Supplementary file 10 — Supplementary file10 (TIF 44847 KB) [file 441_2022_3720_MOESM10_ESM.tif]
