# Supplementary material for: CPNE1 regulates myogenesis through the PERK-eIF2α pathway mediated by endoplasmic reticulum stress
Source: Cell Tissue Res. 2022 Dec 16;391(3):545–60. doi: 10.1007/s00441-022-03720-y (PMC9974702; doi:10.1007/s00441-022-03720-y)
Supplement: Supplementary file 2 — Supplementary file2 (DOCX 13 KB) [file 441_2022_3720_MOESM2_ESM.docx]

Table 1. List of RT-qPCR primers

| Gene | Primer for RT-qPCR | Sequence (5’-3 ’) |
| --- | --- | --- |
| Atrogin1 | Forward | ACGATGTTGCAGCCAAGAAG |
|  | Reverse | GCAGTCGAGAAGTCCAGTCT |
| MuRF1 | Forward | AAGTTTGACGCCCTCTACGC |
|  | Reverse | GCCTTGTTCTGTCTTCCCCA |
| Cpne1 | Forward | GCATTTGGATTTGGAGCCCA |
|  | Reverse | TGATGGGTGCAAAGTTGGTG |
| MyoG | Forward | GTATGAAACATCCCCCTATT |
|  | Reverse | CTCTTACACACCTTACACGC |
| MyoD | Forward | CCTACTACAGTGAGGCGTCC |
|  | Reverse | CTGTTCTGCATCGCTTGAGG |
| Glb1 | Forward | CATGGGGCGTGTGAACTATG |
|  | Reverse | ACGTAAAAGGTGGGGAGTGT |
| Trb3 | Forward | CCATGTCAATGGTGAGAG |
|  | Reverse | TAAGCCCCAGTCGAGTTC |
| Asns | Forward | GACAAAGCGACACAGCTTGA |
|  | Reverse | GTAGCGCCTTGTGGTTGTAG |
| Chop | Forward | TAGTTGGCTGACTGAGGG |
|  | Reverse | GACATGCGGTCGATCAGG |
| Mfn2 | Forward | GCAGCGGGTTTATTGTCTT |
|  | Reverse | GCGGTGCAGTTCATTCTTAT |
| Drp1 | Forward | CCGGTCATCAATAAGCTGCAA |
|  | Reverse | GTGGACCAGCTGCAGAATAAG |
| Gapdh | Forward | ACAGCAACAGGGTGGTGGAC |
|  | Reverse | TTTGAGGGTGCAGCGAACTT |
