# Supplementary material for: CPNE1 regulates myogenesis through the PERK-eIF2α pathway mediated by endoplasmic reticulum stress
Source: Cell Tissue Res. 2022 Dec 16;391(3):545–60. doi: 10.1007/s00441-022-03720-y (PMC9974702; doi:10.1007/s00441-022-03720-y)
Supplement: Supplementary file 5 — Supplementary file5 (DOCX 14 KB) [file 441_2022_3720_MOESM5_ESM.docx]

**Supplementary figures legends**

**Supplementary Figure 1. Satellite cells were isolated by FACS.** Satellite cells (CD31-, CD45-, SCA1-, CD11b-, Itga7+, CD34+) were isolated by FACS.

**Supplementary Figure 2. Base expression of sarcopenia-related genes and *Cpne1* in young and old mouse** **satellite cells (SC)** (a) *Glb1* mRNA level in young and old skeletal muscle satellite cells. *Cpne1*, *Atrogin1*, *MuRF1*, *MyoG* mRNA level (b) in young and old satellite cells. (c) CPNE1 overexpression efficiency is tested by RT-PCR. ^**^*p* < 0.01. (d) β-galactosidase staining in old and young satellite cells and in satellite cells overexpressing CPNE1.
